# Public health round-up

**DOI:** 10.2471/BLT.23.010323

**Published:** 2023-03-01

**Earthquake in the Syrian Arab Republic and Türkiye**

Rescue workers carry a boy from the rubble of a building in Idlib in the Syrian Arab Republic. The building collapsed on 6 February 2023, when a 7.8 magnitude earthquake followed by multiple aftershocks struck south-east Türkiye, close to the border with the Syrian Arab Republic. As of 13 February, some 34 000 people were reported to have died, with the numbers expected to rise in the following days. There was also significant damage to essential infrastructure, including health facilities.

**Figure:**
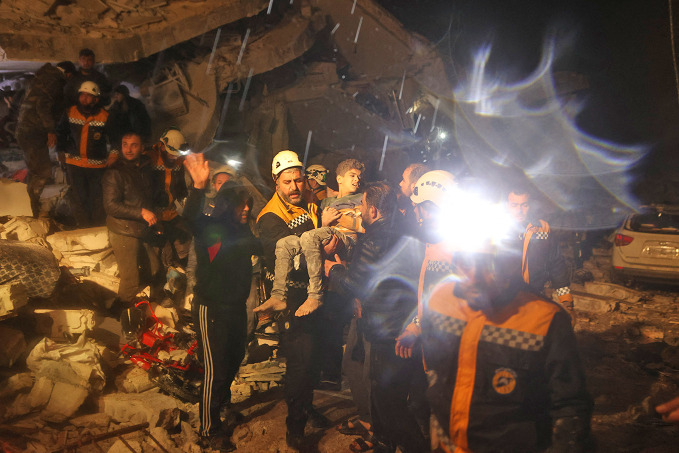
UNICEF/ UN0777953

## WHO’s earthquake response

At the request of the Turkish Government, the World Health Organization (WHO) and other United Nations partners launched a humanitarian response to the earthquake that struck the Syrian Arab Republic and Türkiye on 6 February. 

WHO activated a network of emergency medical teams to provide essential health care for the injured and most vulnerable in the days following the earthquake, and flew 110 metric tonnes of medical supplies out of the WHO Global Logistics Hub located within the International Humanitarian City in Dubai, United Arab Emirates. 

Sufficient to cover the needs of around 400 000 people, the delivery included supplies for urgent surgical interventions and the treatment of anticipated pneumonia cases among survivors, many of whom were left outside in sub-zero temperatures after buildings collapsed.

WHO also released more than 16 million United States dollars (US$) from the Contingency Fund for Emergencies, including US$3 million within hours of the disaster, and, in the Syrian Arab Republic, was able to release prepositioned medical and surgical supplies to 16 hospitals treating survivors of the earthquake in the north-west of the country on day one of the emergency. 

The emergency in the Syrian Arab Republic is being compounded by an ongoing crisis, dating back to 2011, that has left millions exposed to health risks including outbreaks of infectious diseases and inadequate health services including disrupted referral networks. 

http://bit.ly/3lp8CDT

http://bit.ly/3DYKX3h

## Marburg in Equatorial Guinea 

Equatorial Guinea reported its first-ever outbreak of Marburg virus disease. Preliminary tests carried out following the deaths of at least nine people in the country’s western Kie Ntem Province confirmed the presence of the viral haemorrhagic fever.

As of 13 April, 16 other suspected cases with symptoms including fever, fatigue and blood-stained vomit and diarrhoea had also been reported.

Teams were deployed in the affected districts to trace contacts, isolate and provide medical care to people showing symptoms of the disease. WHO also deployed health emergency experts in epidemiology, case management, infection prevention and control, laboratory and risk communication to support the national response efforts and secure community collaboration in controlling the outbreak.

http://bit.ly/3xj2vU2

## Cholera outbreaks in Africa

Africa is witnessing a steep rise in cholera cases amid a global surge. An estimated 26 000 cases and 660 deaths were reported as of 29 January 2023 in the 10 African countries facing outbreaks since the beginning of the year.

The bulk of the new cases and deaths have been recorded in Malawi, which is facing its worst cholera outbreak in two decades, reporting over 600 new cases per day with a continually high case fatality rate (>3%). The outbreak was declared a public health emergency by the Malawi government on 5 December 2022.  

In addition, neighbouring Mozambique has registered a sharp increase in cases and alerts since mid-December 2022, with cases reported from five provinces, including provinces bordering Malawi. On 26 January 2023, Zambia notified WHO of a cholera outbreak in the eastern province bordering Malawi and Mozambique. There remains a high risk of spread to other countries in the region, including the United Republic of Tanzania and Zimbabwe. 

In East Africa, Ethiopia, Kenya and Somalia are responding to outbreaks amid a prolonged and harsh drought that has left millions of people in dire need of humanitarian assistance. 

http://bit.ly/3xcHKJK

http://bit.ly/3YJRg2H

## Contaminated medicine alert

Between October 2022 and January 2023, seven WHO Member States reported incidents of over-the-counter cough syrups for children being sold with high levels of diethylene glycol.

More than 300 children were reported to have died as a result of ingesting the syrup in three of the countries concerned (Gambia, Indonesia and Uzbekistan). Most were young children under the age of five. 

On 23 January, WHO issued medical product alerts to the national health authorities of all 194 WHO Member States requesting the detection and removal of contaminated medicines from circulation, increased supply chain surveillance, immediate notification to WHO of substandard products found, and public information campaigns. Because these were not isolated incidents, WHO called on various stakeholders engaged in the medical supply chain to take immediate and coordinated action.

http://bit.ly/3YEQYdk

## Boosting access to new tuberculosis vaccines

WHO Director-General, Tedros Adhanom Ghebreyesus, announced plans to establish a tuberculosis Vaccine Accelerator Council to facilitate the licensing and use of effective, novel tuberculosis vaccines, if and when they become available. 

Speaking at a high-level panel on tuberculosis at the World Economic Forum on 17 January, Director-General Tedros explained that the council will seek to catalyze high-level alignment between funders, global agencies, governments and end-users to make sure that innovative products reach the people who need them.

“One of the most important lessons from the response to the COVID-19 pandemic is that innovative health interventions can be delivered fast if they are prioritized politically and financed adequately,” he said.

http://bit.ly/3JV1UzB

## Germany supporting WHO

Germany’s Federal Ministry of Health announced a 130 million euros (US$140 million) contribution to WHO for 2023, reinforcing Germany’s role as a global health leader and one of WHO’s strongest supporters. The announcement was made on the sidelines of the 152nd session of WHO’s Executive Board which took place at WHO’s Geneva headquarters between 30 January and 7 February.

Germany was WHO’s largest overall contributor in the 2020-2021 biennium, and second-largest in 2022. The country has also been the top donor to the ACT-Accelerator, a global collaboration designed to support the rapid development, production and equitable distribution of COVID-19 vaccines, tests and treatments. The contribution will support WHO in priority areas including antimicrobial resistance, health emergencies, health systems strengthening and WHO reforms. 

http://bit.ly/3DWXx3k

## Missing the trans fat target

Five billion people globally remain unprotected from harmful industrially produced trans fat despite a target to eliminate the product from human consumption by 2023, called for by WHO in 2018.

Trans fat is commonly found in packaged foods, baked goods, cooking oils and spreads, and is estimated to be responsible for up to 500 000 premature deaths from coronary heart disease each year.

According to a status report published by WHO on January 23 that assesses global progress towards the 2023 target, 43 countries have introduced best-practice anti-trans fat policies that are helping to protect some 2.8 billion people globally. While this clearly represents progress, some 5 billion people remain exposed to the product. 

“Trans fat has no known benefit, and huge health risks that incur huge costs for health systems,” said Director-General Tedros at the report’s launch, adding that it was time to get rid of the product, “once and for all.”

http://bit.ly/3HOBT27

**Cover photo**

A place of worship in Gaziantep in Türkiye that was devastated by the earthquake that hit the country and neighbouring Syrian Arab Republic on 6 February 2023.

**Figure:**
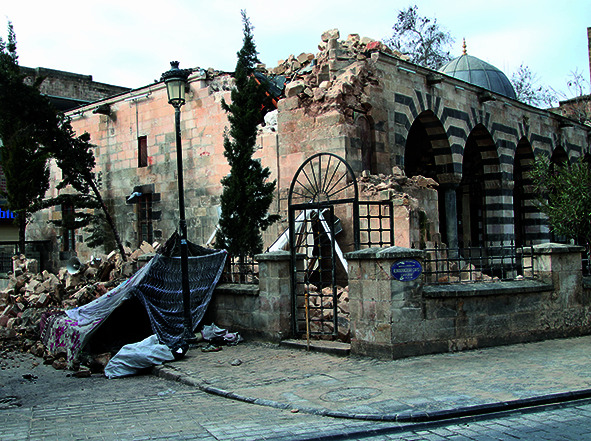
WHO/MAHER ABDULAZIZ/Arete/DEC

## Progress on neglected tropical diseases

The number of people requiring neglected tropical disease (NTD) interventions fell by 80 million between 2020 and 2021, and eight countries were certified or validated as having eliminated one NTD in 2022 alone. As of December 2022, 47 countries had eliminated at least one NTD and more countries were in the process of achieving this target.

This is according to the *Global report on neglected tropical diseases* *2023* that was released on 30 January, highlighting the progress made and challenges faced in delivering NTD care worldwide, against a backdrop of COVID-19-related disruptions.

The report notes that the advances made in 2021-2022 build on a decade of significant progress that includes one billion people being treated for NTDs each year between 2016 and 2019 through mass treatment interventions, and 25% fewer people requiring interventions against NTDs in 2021 than in 2010.

http://bit.ly/3HQXAP3

**Looking ahead**

6 – 17 March. 67^th^ Session of the United Nations Commission on the Status of Women. United Nations Headquarters, New York. http://bit.ly/3jQqtTJ

27 – 31 March. Global meeting on skin-related neglected tropical diseases. WHO Geneva HQ. http://bit.ly/3DLSAdm

3 – 5 April 2023. Fifth Global Forum on Human Resources for Health. Geneva, Switzerland. http://bit.ly/3kjKQsm

